# Latest pharmaceutical approaches across the spectrum of heart failure

**DOI:** 10.1007/s10741-024-10389-8

**Published:** 2024-02-13

**Authors:** Dimitrios Bismpos, Jan Wintrich, Julian Hövelmann, Michael Böhm

**Affiliations:** 1 Department of Internal Medicine III, Cardiology, Angiology and Intensive Care Medicine, University Hospital, Saarland University, Homburg, Saar, Germany; 2 Department of Internal Medicine II, Cardiology and Angiology, Marien Hospital Herne, University Clinic of the Ruhr University, Bochum University, Herne, Germany

**Keywords:** Heart failure, Heart failure with reduced ejection fraction (HFrEF), Heart failure with mildly reduced ejection fraction (HFmrEF), Heart failure with preserved ejection fraction (HFpEF)

## Abstract

Despite major advances in prevention and medical therapy, heart failure (HF) remains associated with high morbidity and mortality, especially in older and frailer patients. Therefore, a complete, guideline-based treatment is essential, even in HF patients with conditions traditionally associated with a problematic initiation and escalation of the medical HF therapy, such as chronic kidney disease and arterial hypotension, as the potential adverse effects are overcome by the overall decrease of the absolute risk. Furthermore, since the latest data suggest that the benefit of a combined medical therapy (MRA, ARNI, SGLT2i, beta-blocker) may extend up to a LVEF of 65%, further trials on these subgroups of patients (HFmrEF, HFpEF) are needed to re-evaluate the guideline-directed medical therapy across the HF spectrum. In particular, the use of SGLT2i was recently extended to HFpEF patients, as evidenced by the DELIVER and EMPEROR-preserved trials. Moreover, the indication for other conservative treatments in HF patients, such as the intravenous iron supplementation, was accordingly strengthened in the latest guidelines. Finally, the possible implementation of newer substances, such as finerenone, in guideline-directed medical practice for HF is anticipated with great interest.

Heart failure (HF) remains one of the major causes of morbidity and mortality worldwide, as its prevalence is rising annually. The last few years have produced major advances in prevention and medical therapy, making a complete, guideline-based treatment a necessity, even in patients with comorbidities traditionally associated with a complicated initiation and up-titration of the medical HF therapy. The aim of this study was to provide an overview on the available pharmaceutical options across the ejection fraction spectrum while providing insight on the management of patients with comorbidities, according to the latest trials and published guidelines [1–3].

## Therapy algorithm for HFrEF

**Fig. 1:**
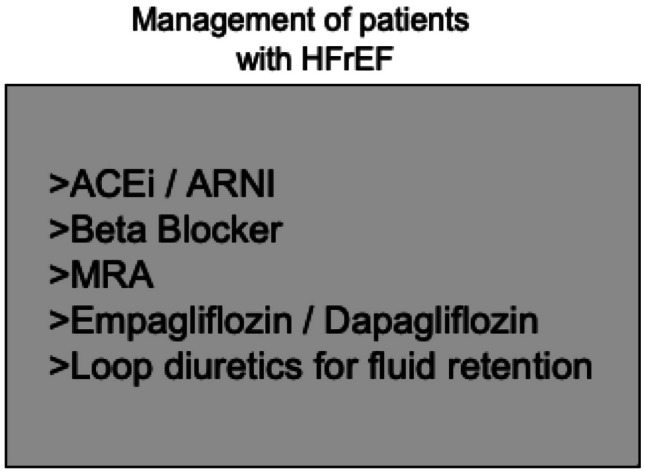
Pharmacological treatment for patients with HFrEF according to the latest ESC algorithm [2]

**Fig. 2:**
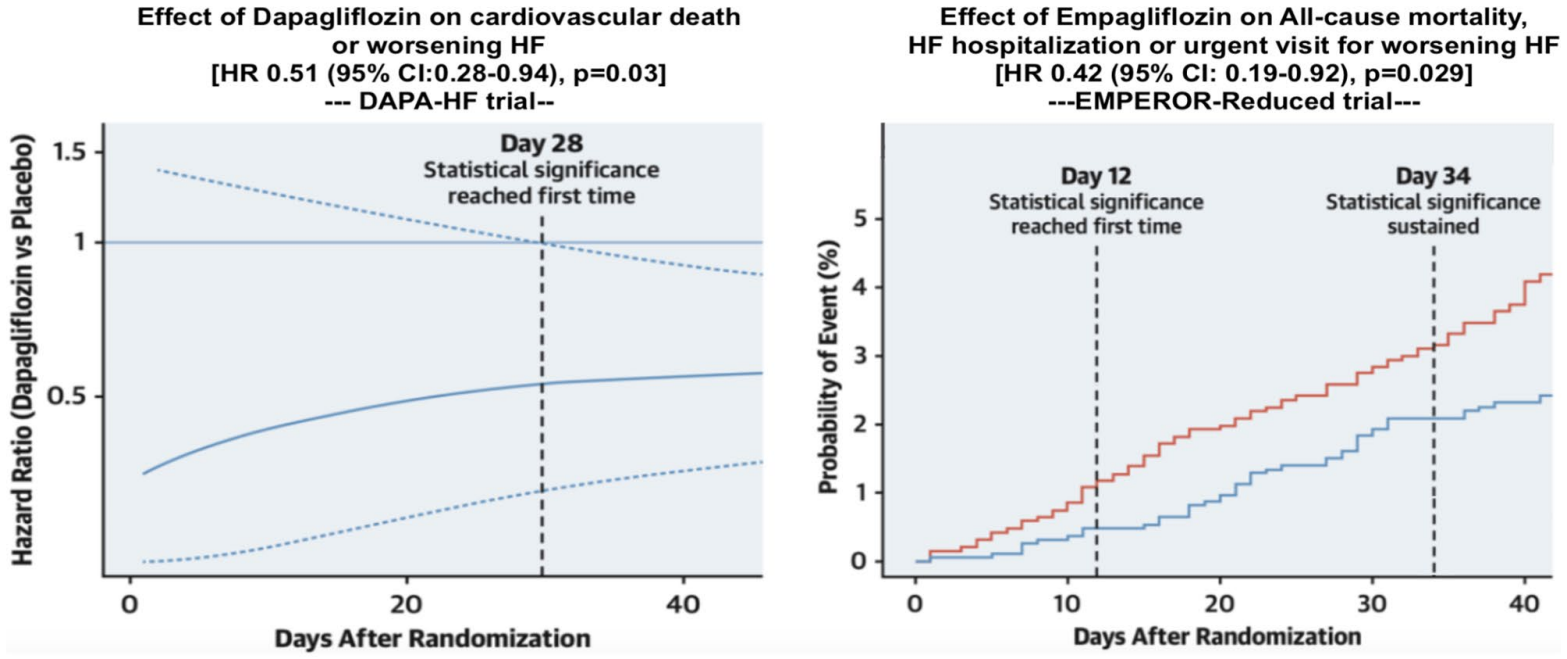
Time to significant treatment effect for dapagliflozin (left) and empagliflozin (right) in patients with HFrEF, modified after Rao et al. [9]

**Fig. 3:**
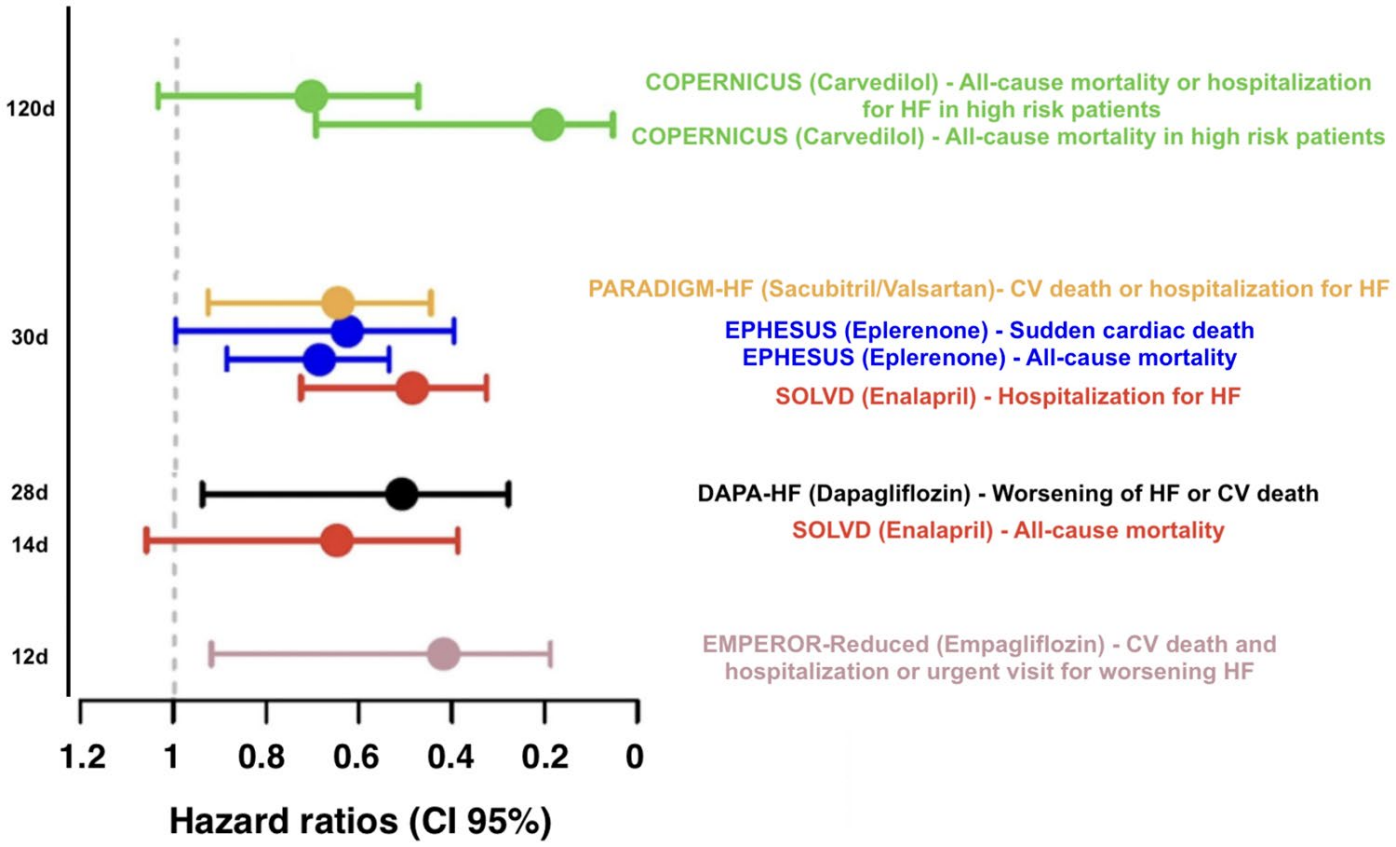
Time to significant treatment effects in the most major heart failure drug clinical trials, modified after Abdin et al. [10]

According to the treatment algorithms of the European Society of Cardiology (ESC) [2], the American College of Cardiology and the American Heart Association and American Heart Failure Association [3], an immediate initiation of treatment for heart failure with reduced ejection fraction (HFrEF) is imperative. Α complete guideline-directed medical therapy (GDMT) should comprise the four following substances: (1) angiotensin-converting enzyme inhibitors (ACEi) or angiotensin receptor-neprilysin inhibitors (ARNI), (2) beta blockers, (3) mineralocorticoid receptor antagonists (MRA), and (4) sodium-dependent glucose co-transporter 2-inhibitors (SGLT2i) (Fig. 1). While either ACEi or ARNI can be used, the AHA guidelines prioritize the use of ARNI as the inhibitor of choice of the renin-angiotensin system in patients with HFrEF in NYHA II-III stadium, while emphasizing their de novo use in patients with acute HF before discharge [3]. Conversely, the ESC guidelines also recommend the use of ARNI (class I), albeit as a replacement for ACEi, while providing an IIb recommendation for their use in the de novo HF [2, 4]. In the case of ACEi/ARNI intolerance, angiotensin II type 1 receptor blockers (ARB) serve as an alternative [2, 3]. This paradigm shift stands in contrast to the stepwise up-titration of drug therapy as recommended by the previous 2016 ESC guidelines on HF [5] and was derived from the rapid onset of significant treatment effects with sacubitril/valsartan in the PARADIGM study [6] as well as findings from the DAPA-HF [7] and the EMPEROR-Reduced Trial [8], in which the effect for dapagliflozin became significant after 28 days and for empagliflozin after only 12 days [9] (Fig. 2). Accordingly, a similar early onset of positive treatment effects has been observed with all agents such as ACEi in the SOLVD study, MRA in the EPHESUS study, and beta-blockers in the COPERNICUS study [10] (Fig. 3). Furthermore, it has been recently shown that postponing heart failure (HF) treatment with ACEi, beta-blockers, and MRA can lead to an increase of 1-year mortality up to a total of 12.2% [11]. Noteworthy, the latter study did not take into account that the most contemporary drugs (i.e., ARNI, SGLT2i) are associated with a significant add-on effect, which likely leads to an underestimation of the impact. A recent analysis from the Swedish HF Registry [12] confirmed the superiority of ARNI over ARB/ACEi, demonstrating a real-world, significant 23% relative risk reduction in all-cause mortality (HR 0.77 [0.63 – 0.95]). Overall, if all substances are administered completely, the event-free survival in a patient aged 80 and 55–60 years can be improved by about 2, 7 and 8, 3 years, respectively [13]. However, availability and access to GDMT (e.g., in low-to-middle income-countries) sadly remains an important prognostic factor even to this day [14].

## Therapy algorithm for HFpEF/HFmrEF

Until recently, heart failure with preserved ejection fraction (HFpEF) constituted a therapeutic problem, since the studies carried out in this patient group failed to show a benefit for ACEi, ARB, digitalis, and beta blockers. Thus, the dysregulation of the renin–angiotensin–aldosterone-systems (RAAS) appears to be of minor importance in HFpEF as opposed to HFrEF [15]. However, the term HFpEF was first introduced in the 1990s and referred to a left ventricular ejection fraction (LVEF) of ≥ 40%. Therefore, the majority of “HFpEF trials”' included both patients with HFpEF (LVEF ≥ 50%) and heart failure with mildly-reduced ejection fraction (HFmrEF, LVEF 41–49%), according to the current ESC guidelines. In a large pooled analysis of two prospective observational studies, clinical characteristics and outcomes in patients with HFmrEF resembled more closely those with HFrEF than HFpEF [16]. Thus, these patients seem to derive dose-dependent benefits from pharmacological therapies on a par with patients with HFrEF [16]. In accordance, post-hoc analyses suggested significant treatment effects for ACEi, ARB, ARNI, and MRA in patients suffering from HFmrEF. Subsequently, the 2021 as well as the focused-2023 ESC guidelines recommend treatment with the aforementioned substances with class IIb indication (level of evidence C) [1–3, 17]. Moreover, to underline the differences between HFmrEF and HFpEF, recent consensus statements favored the term “heart failure with normal ejection fraction” in HF patients with LVEF ≥ 50% [18].

Importantly, the 2021 ESC guidelines did not consider findings from both the DELIVER and the EMPEROR-Preserved trial, which had not been published during the development of the guidelines. These randomized, placebo-controlled trials studied the effects of dapagliflozin and empagliflozin in HFpEF and HFmrEF patients. In both trials, the primary endpoint of cardiovascular mortality and HF events was significantly reduced in patients treated with SGLT2i, regardless of concomitant diabetes. Moreover, therapy with SGLT2i improved quality of life measures. [19–21]. Notably, recent meta-analyses have documented equally significant treatment effects in HFmrEF and HFpEF patients treated with empagliflozin as well as those with dapagliflozin [22, 23]. Furthermore, the onset of a significant treatment effect was 18 days for empagliflozin [23] and 30 days for dapagliflozin [24], which reinforces the idea of a prompt initiation of the appropriate substances, even in patients with HFpEF or HFmrEF. As a result, the findings of the DELIVER and the EMPEROR-Preserved trials were incorporated into the latest focused-ESC guidelines with Class I indication for HFmrEF and HFpEF [1].

**Fig. 4:**
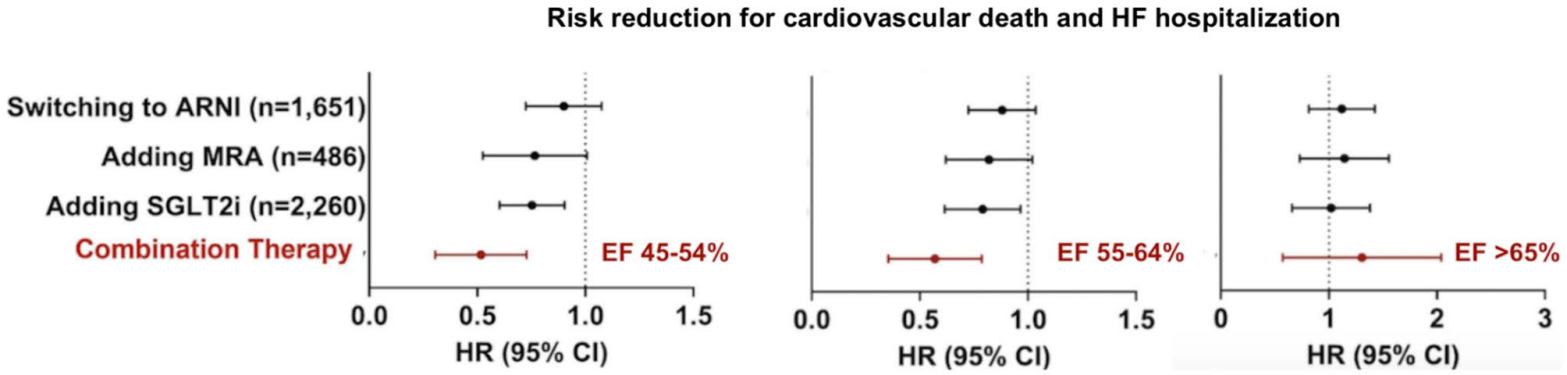
Estimated treatment effects of a combined medical therapy by LVEF category, modified after Vaduganathan et al. [25]

Finally, recent subgroup analyses suggest that a combination therapy with MRA, ARNI, and SGLT2i may have positive cardiovascular effects on patients with LVEF up to 55% [25] (Fig. 4). The landmark PARAGON-HF failed to show a significant benefit of sacubitril-valsartan in patients with HF with EF ≥ 45% regarding the composite outcome of total hospitalizations for HF and cardiovascular death. However, a modest though statistically not significant lower rate of hospitalizations for HF was observed. Furthermore, when examining the subgroup of patients with EF between 45 and 57%, a significant benefit was suggested [26]. In the recently published PARAGLIDE-HF trial, sacubitril/valsartan led to a greater reduction in plasma NT-proBNP levels in comparison to valsartan alone in patients with an EF > 40% and a recent worsening HF event [27]. A pooled analysis of PARAGLIDE-HF and PARAGON-HF, which included participants with mildly reduced or preserved LVEF (> 40% in PARAGLIDE-HF and ≥ 45% in PARAGON-HF), demonstrated that sacubitril/valsartan reduced the primary composite endpoint of worsening HF events (including first and recurrent HF hospitalization) and cardiovascular death significantly compared to valsartan (RR, 0.86; 95% CI, 0.75–0.98; NNT, 14) [28]. However, further trials are needed to examine the net clinical benefit on patients with HFmrEF and HFpEF, respectively.

## Treatment of iron deficiency

Iron deficiency constitutes a frequent comorbidity in heart failure patients [29], with evidence suggesting that iron supplementation may have cardioprotective effects. More specifically, in the AFFIRM-AHF trial, among patients with acute HF and iron deficiency, intravenous ferric carboxymaltose was associated with a reduction in cardiovascular death and hospitalization [30]. This result was consistent with a meta-analysis of seven randomized trials, driven mainly by a substantial reduction in HF hospitalizations [31]. Accordingly, the European and American guidelines [2, 3] already recommended to measure the iron status of every patient with chronic HF (class I), using the ferritin levels (100–299 ng/ml) and transferrin saturation (< 20%) as indicators of absolute or functional iron deficiency, as well as considering intravenous iron repletion (class IIa). The IRONMAN study, which was recently published, reinforces the strategy of iron repletion for a broad range of patients with heart failure, reduced LVEF ≤ 45%, and iron deficiency [32]. Of note, a meta-analysis of ten randomized trials showed that the beneficial cardiovascular outcome of intravenous iron infusion in patients with HF and iron deficiency was consistent among patients with and without anemia [33]. Despite this knowledge and the reported high prevalence of iron deficiency in HF patients (up to 50%), iron testing is carried out far too seldom, resulting in an even less frequent initiation of treatment [34]. Consequently, after the publication of the latest randomized controlled trials, the recommendation for intravenous iron repletion was recently strengthened (class I to alleviate symptoms and improve quality of life, class IIa to reduce the risk of HF hospitalization) [1].

## Newer substances

**Fig. 5:**
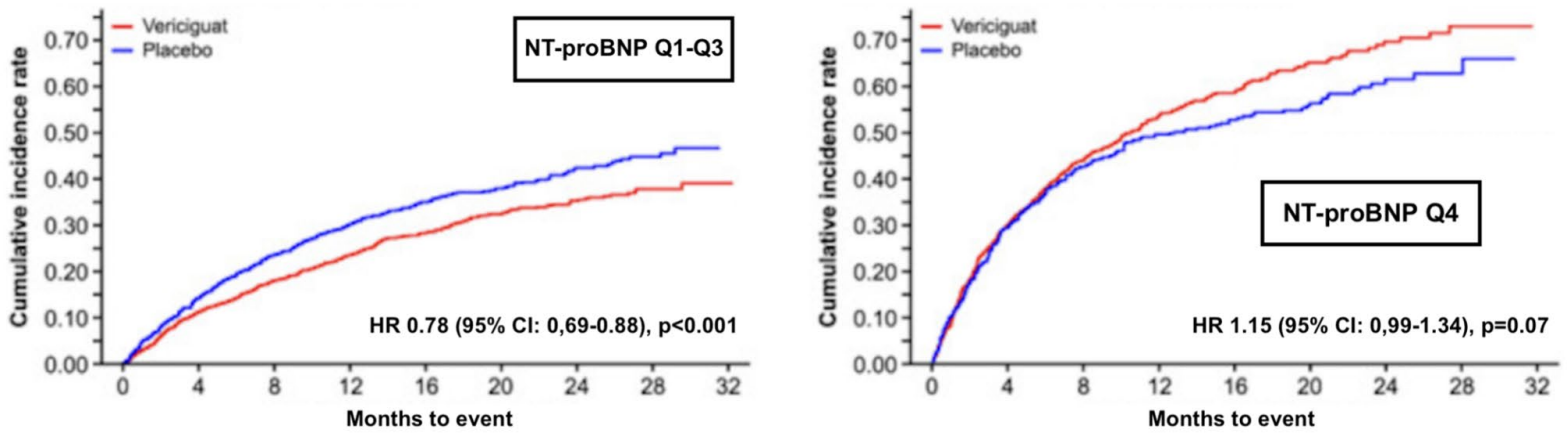
Effect of vericiguat on cardiovascular outcomes according to NT-proBNP levels, modified after Senni et al. [36]

One promising substance in the treatment of HF is vericiguat, a guanylate-cyclase-activator which accelerates the formation of cGMP in the heart and vasculature [35]. Recently, the VICTORIA trial demonstrated significantly lower rates for cardiovascular death and HF hospitalizations among patients with symptomatic HF (NYHA II-IV) and a reduced LVEF of ≤ 45% [36]. Considering the particularly high event rate in this group of patients, who were often hospitalized with an acute decompensation of HF and already receiving the maximum evidence-based medical treatment, vericiguat led to an absolute event-rate reduction of 4.2 events per 100 patient-years. This absolute risk reduction compares to those seen with ARNI and SGLT2i [37]. That said, the added effect of vericiguat in patients already on GDMT remains to be clarified in the future, as patients with ARNI and SGLT2i were not properly represented in the trial. Of note, treatment effects did not differ among prespecified subgroups (e.g., different NYHA classes at baseline), though it must be noted that only a small fraction of the patient population suffered from advanced HF (NYHA IV). In this direction, according to a post-hoc analysis of the VICTORIA trial [37], patients with very high levels of NT-proBNP (here defined as levels over the 75th percentile; meaning > 5314 pg/ml) suffer probably from a far too advanced and/or destabilized HF as well as many comorbidities to derive benefit from vericiguat (Fig. 5). In these cases, an optimization of the volume status before initiation of the therapy should be strongly considered.

Another substance of interest is omecamtiv-mecarbil, which augments cardiac contraction through an increase in LV systolic ejection time, by selectively binding to cardiac myosin, leading to an improvement of systolic cardiac function [38]. Recently, the GALACTIC-HF-Trial showed a reduction of cardiovascular events by 8% in HFrEF patients (LVEF ≤ 35%) [39]. Secondary analyses revealed a strengthened impact on patients with severe heart failure (i.e., LVEF < 30% in sinus rhythm, NYHA Class III-IV, hospitalization due to HF in the previous 6 months), supporting a potential role of omecamtiv-mecarbil among patients for whom current treatment options are limited [40]. However, it must be noted that omecamtiv-mecarbil failed to significantly improve exercise capacity in a group of patients with chronic HFrEF [41]. Nonetheless, the use of omecamtiv-mecarbil in patients with HFrEF was recently declined by the FDA, as more clinical trials are needed to establish its effectiveness for the treatment of HFrEF.

The beneficial impact of SGLT2i on patients with diabetes and HF can be expanded to other substances, such as the non-selective SGLT2i sotagliflozin, as shown in the SOLOIST-WHF trial. By delaying intestinal glucose absorption through an additional gastrointestinal SGLT1 inhibition, sotagliflozin can reduce postprandial glucose levels [42]. In this direction, it was demonstrated that the initiation of therapy with sotagliflozin before or shortly after discharge can lead to a reduction in cardiovascular morbidity and hospitalizations for HF in patients with worsening HF and type 2 diabetes [42]. In fact, a large meta-analysis with HFrEF patients showed that non-selective SGLT2i such as sotagliflozin may be superior to highly selective SGLT2i in terms of HF outcomes [43]. More trials investigating the role of receptor-selectivity of SGLT2i in HF treatment are warranted.

## Patients with comorbidities

Chronic HF is accompanied by numerous cardiac and non-cardiac comorbidities, which equally affect management and prognosis. Despite major advances in HF treatment and the overall emphasis on prevention, the comorbidity burden remains high in patients with HF (mean 3.9 comorbidities per patient) and is associated with a worse outcome [44]. Comorbidities with the greatest prevalence are chronic kidney injury, anemia, diabetes, and obesity, affecting most frequently patients with HFpEF [45]. These results can be primarily attributed to the older age of HFpEF patients. Accordingly, since each comorbidity contributes to the mortality rate, patients with HFpEF and HFmrEF are more frequently affected by non-cardiovascular mortality than HFrEF patients [45, 46]. At the same time, comorbidities such as arterial hypertension, chronic kidney injury, and old age often pose a barrier to therapy initiation, continuation, and escalation.

### Arterial hypotension

A reverse J-curve relationship seems to exist between mortality and blood pressure in patients with HF, regardless of LVEF [47]. A reduction in blood pressure, whether due to the progression of HF or the coexisting diseases, leads to a substantial increase in absolute risk. On the other hand, hypotension may result in complete intolerance of most HF drugs. Thus, treating physicians may withhold or discontinue HF treatment due to the concern of symptomatic hypotension [47]. The question that arises is whether HF drugs, most of which lower blood pressure, remain of prognostic importance even in hypotensive conditions. In the PARADIGM-HF Trial, ARNI led to a greater decrease in systolic blood pressure in comparison to enalapril, while attaining a consistent cardiovascular benefit across the blood pressure spectrum [48]. Hence, compared to patients with high blood pressure (systolic blood pressure ≥ 140 mmHg), therapy with ARNI led to a greater absolute risk reduction in patients with low blood pressure (systolic blood pressure < 110 mmHg) [48]. Analogously, in HFrEF patients, Dapagliflozin [49] and empagliflozin [50] provided a reduction of the relative risk for cardiovascular outcomes irrespective of blood pressure. Of note, administration of SGLT2i slightly reduced the blood pressure solely in patients with relatively high values (> 130 mmHg). In accordance, a post-hoc analysis of the EMPEROR-Preserved trial [21] has shown that empagliflozin treatment effects in HFpEF patients were not moderated by systolic blood pressure as well [51]. In principle, it has been demonstrated that the adverse effects associated with hypotension are overcome by the overall decrease of the absolute risk related to the HF treatment [48–51]. This argument was supported by a large analysis of the Swedish HF Registry, where a maximal GDMT in HFrEF patients with hypotension and impaired renal function was associated with an improved survival [52].

### Age

In older patients, up-titration of medical HF therapy may be difficult due to adverse effects and non-adherence. Thus, treating physicians may be less committed to maximize the treatment. Older patients, especially those over 80 years, rarely (25%) receive the recommended triple therapy (ACEi/ARNI, beta-blocker, MRA) [53]. Frailty in old age seems to especially derail the initiation or up-titration of HF therapy, with 61% of HFrEF patients receiving a sub-optimal medical treatment [54]. However, concerns were raised that the efficacy of the medical therapy was diminished in elderly HF patients, particularly in those with HFpEF [55]. That said, Empagliflozin improved outcomes in patients with HFpEF regardless of age, while also improving the quality of life, without an increase of the serious adverse events in the elderly [56]. In fact, the absolute risk reduction in patients > 75 years was even slightly albeit not significantly higher than in younger patients [56]. Moreover, in a recent meta-analysis, the treatment effects of all HF drugs were found to be stable with age [57]. These findings emphasize the importance of HF treatment in elderly patients since the absolute benefit is maximized in this age group [56, 57].

#### Obesity

HFpEF is frequently associated with obesity and is thereby linked with changes in metabolism [58]. Obese patients actually represent the majority of HFpEF patients [59] and are burdened by more severe symptoms and impaired quality of life [60, 61]. While smaller, observational studies have hinted at the possible benefit of weight loss in cardiac function [62], until recently the role of pharmacotherapy for weight loss in HFpEF had not been studied. The STEP-HFpEF study investigated the role of the glucagon-like peptide-1 receptor agonist (GLP-1RA) semaglutide in patients with the obesity phenotype of HFpEF, showing improvements in exercise function as well as a significant weight loss compared to placebo [63]. Similarly, the STEP-HFpEF DM study (NCT04916470) aims to examine the role of semaglutide in the same set of patients who also suffer from type 2 diabetes.

### Chronic kidney disease

Chronic kidney disease (CKD) is the most common comorbidity in HF, affecting approximately half the patients, particularly those with HFpEF [34, 64]. Regardless of the type of HF (HFrEF, HFmrEF, HFpEF), mortality from HF inversely correlates with the decreasing renal function [64]. Despite the elevated risk, patients with comorbid CKD are often not optimally treated, even when the evidence-based medical therapy is not contraindicated by kidney dysfunction [64]. However, the pharmacokinetic limitations of many HF drugs have to be acknowledged in HF patients with comorbid CKD, which may hamper the optimization of treatment.

Nevertheless, some HF drugs, and in particular SGLT2i, are associated with nephroprotective effects. For instance, SGLT2i has been shown to delay the decrease in estimated glomerular filtration rate (eGFR) over time [7, 8]. By vasoconstriction of the vas afferens SGLT2i reduces the effective filtration pressure in the glomeruli and stimulates the tubuloglomerular feedback mechanism, which results in nephroprotection and decreased microalbuminuria [65]. These effects were demonstrated to be independent of the baseline kidney function, presence of diabetes [66], age, type of HF (HFrEF, HFmrEF, HFpEF) as well as blood pressure [56]. Of note, the administration of SGLT2i is associated with an initial temporary decrease in eGFR [65–68], which is aggravated by an already impaired kidney function [69] as well as the common use of diuretics in HF patients [70]. Interestingly, in a post-hoc analysis of the DAPA-HF trial, a pronounced eGFR dip (> 10%) at the beginning of SGLT2i treatment was associated with a decreased risk of the primary endpoint of worsening HF or cardiovascular death [68].

As a result of these positive post-hoc findings, two large studies were recently conducted on nephroprotection with SGLT2i treatment in patients with impaired renal impairment and albuminuria/proteinuria. In the DAPA-CKD-Trial, patients with CKD already on nephroprotective therapy with ACEi/ARB had a significantly lower risk for cardiovascular events as well as for a progression of the kidney disease when receiving dapagliflozin, regardless of the presence of diabetes mellitus [70]. Similarly, the EMPA-KIDNEY-trial studied the treatment effects of empagliflozin in patients with pronounced CKD or with significant albuminuria, revealing a decrease in renal endpoints and a low rate of cardiovascular death [71]. Subsequently, the use of SGLT2i in patients with CKD and type 2 diabetes is recommended in the latest focused ESC guidelines to reduce the risk of HF hospitalization or cardiovascular death (class I indication) [1].

**Fig. 6:**
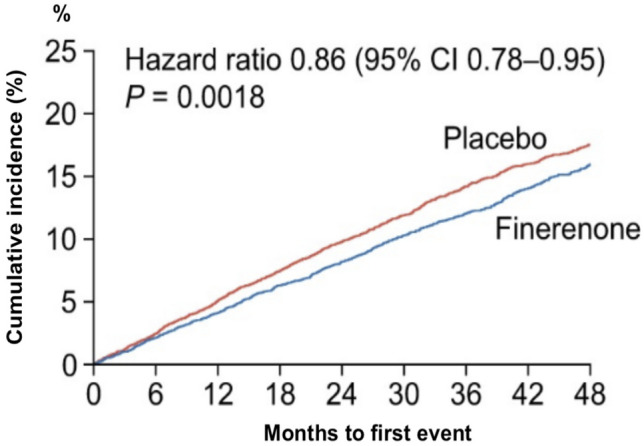
The effect of finerenone on cardiovascular outcomes in patients with CKD and DMT2, modified after Agarwal et al. [75]

Another promising substance, which has so far been primarily examined as a nephroprotective therapy, is the selective, non-steroidal MRA finerenone. In contrast to the conventional MRAs, finerenone has a more specific effect on the heart and kidneys, while bearing a lower risk of hyperkalemia and hypotension [72]. To test the hypothesis that finerenone can slow the progression of CKD and reduce cardiovascular morbidity and mortality, two major trials were recently conducted, exhibiting a decrease of cardiovascular outcomes (death, myocardial infarction, stroke, hospitalization for heart failure) as well as of the progression of the renal disease [73–75]. Because a substantial ratio of patients (40%) was included solely on the basis of albuminuria (albumin-to-creatinine ratio > 300) while having a normal eGFR, screening for albuminuria is vital to identify all patients at risk [75] (Fig. 6). Patients with reduced ejection fraction were excluded from the aforementioned trials; however, it was demonstrated that finerenone use in patients with CKD and type 2 diabetes mellitus prevents hospitalizations due to HF [75]. Correspondingly, the use of finerenone in patients with type 2 diabetes and CKD is recommended in the latest 2023 focused update of the ESC guidelines to reduce the risk for HF hospitalization [1]. The FINEARTS-HF trial (NCT04435626) on the effect of finerenone on cardiovascular death and HF hospitalizations on primarily HF patients with EF > 40% is currently in the recruitment phase.

Finally, the DIAMOND trial demonstrated that the concurrent use of MRA and patiromer in patients with HFrEF and renin–angiotensin–aldosterone-system-inhibitor (RAASi)-related hyperkalemia was associated with significantly lower levels of potassium in serum and hyperkalemia-related adverse events as well higher, guideline-directed RAASi use [76]. This benefit was present and actually even more prevalent in patients with CKD, who already are predisposed to hyperkalemia, which could be of use for the treatment of CKD patients with HFrEF.

While eGFR measured with creatinine has been largely used in the aforementioned trials to stratify patients with HF and impaired renal function as well as to guide the initiation and up-titration of GMDT, a discrepancy between eGFR measured with cystatin C and creatinine has already been described [77, 78]. The use of cystatin C alone or in combination with creatinine is predictive of death as well as end-stage renal disease [77]. Accordingly, a post-hoc analysis of the PARADIGM-HF trial revealed that the occurrence of worsening HF was associated with a more pronounced decline in kidney function when assessed by eGFR measured with cystatin C [78], raising questions about the optimal assessment of the renal function in patients with HF.

## Venous congestion

Cardiorenal syndrome encompasses a variety of disorders involving both the heart and the kidneys. Decreased renal perfusion due to chronic HF (type 2 cardiorenal syndrome) with activation of the neuroendocrine system leads to increased retention of sodium, which in turn causes a venous congestion, with adverse effects on both the heart and the kidneys [79]. Another described mechanism is fluid retention due to acute HF (type 1 cardiorenal syndrome), causing a congestion in the kidneys, and thus further impairment of the renal function [80].

**Fig. 7:**
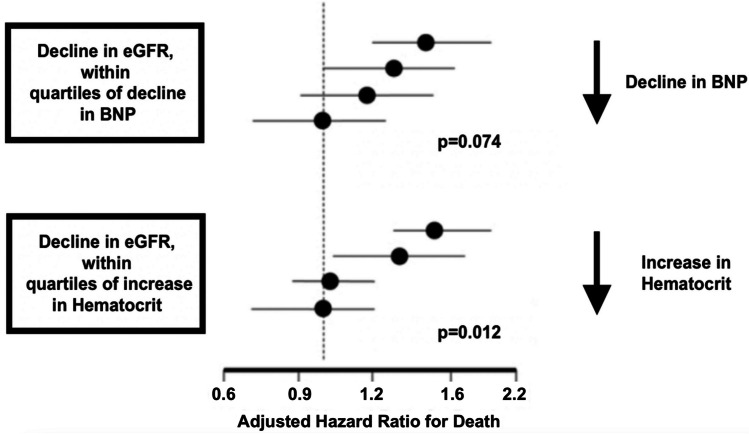
Decongestion as measured by a decline in b-type natriuretic peptide (BNP) and hemoconcentration as measured by an increase in hematocrit is associated with decreased risk of death, modified after McCallum et al. [82]

Contrary to the long-held opinion, the acute decline in eGFR during a diuretic therapy is not associated with increased mortality and persistent kidney function impairment, as long as there is evidence of decongestion [81]. This can be objectively measured by declines in BNP, NT-proBNP, and weight or by an increase in hematocrit, albumin, and total protein [82] (Fig. 7). In this respect, the rapid decongestion of the kidneys plays a crucial role in the treatment of acute decompensated HF.

Furthermore, initiation of a sequential nephron blockade is an important therapeutic approach to enhance the diuretic response. In this context, the sequential nephron blockade with the carbonic anhydrase inhibitor acetazolamide, that reduces proximal tubular sodium reabsorption, in addition to loop diuretic therapy has been evaluated in the ADVOR trial. In this trial, 510 patients with acute decompensated HF were randomly assigned to standardized intravenous loop diuretic therapy plus acetazolamide versus standardized intravenous loop diuretic therapy plus matching placebo. It was shown that the additional administration of acetazolamide resulted in a faster decongestion within 3 days after treatment initiation, without worsening the kidney function or affecting the blood pressure [83]. Similarly, in the EMPAG-HF trial, the early addition of empagliflozin to standard diuretic therapy was associated with increased urine output and a more pronounced decrease in NT-proBNP. These findings resulted from an enhanced sodium and water excretion in the proximal tubule [84]. Moreover, a recently published meta-analysis demonstrated that SGLT2i therapy in addition to conventional HF treatment resulted in higher volumes of diuresis with a lower dose of loop diuretics and led to a reduction in cardiovascular events [85]. Finally, a classic sequential nephron blockade with the inclusion of a thiazide diuretic also led to a faster decongestion within 72 and 96 h compared to loop diuretic therapy only. In addition, there was a trend towards decreased risk of rehospitalization in patients treated with both hydrochlorothiazide and furosemide [86]. However, this strategy can be accompanied by an increased risk for an impairment of renal function [86].

## Steps after discharge

According to the latest guidelines [1, 2], acutely decompensated patients should not be discharged until fully recompensated, i.e., without residual signs of congestion or edema. Patients with residual congestion at discharge had significantly less favorable cardiovascular outcomes (death or rehospitalization), especially in those with worsened renal function and old age [87]. However, a complete recompensation at discharge is only established in 30–50% of decompensated HF patients, as demonstrated by the register of the European Society of Cardiology (ESC-EORP-HFA, [88]). Moreover, recent trials underline the importance of initiating a proper HF therapy after decompensation as soon as possible, while the patients are still treated in the hospital. For instance, in the EMPULSE trial, the early administrations of empagliflozin (24 h after hemodynamic stabilization) in patients hospitalized with acute HF resulted in a survival benefit, while also reducing the rate of rehospitalizations and improving the symptoms [89]. Overall, the treatment with empagliflozin produced a 26% win ratio, a finding that was generally consistent across all specified subgroups, including patients with HFrEF or HFpEF as well as with or without type 2 diabetes mellitus [89]. Similarly, in the STRONG trial, a rapid complementation and up-titration of the full guideline-recommended HF therapy were associated with a decreased risk of the composite endpoint of all-cause mortality and HF hospitalizations (risk ratio 0,66 (95% CI 0.50–0.86)) [90, 91]. Furthermore, the early HF therapy is associated with improved quality of life [91]. Therefore, high-intensity care for initiation and up-titration of pharmaceutical therapy, as well as a close follow-up is recommended and was for that reason incorporated into the latest focused-ESC guidelines [1]. This strategy sadly remains challenging in clinical practice, as evidenced by the results of the CHAMP-registry: over a 1-year long follow-up period, less than 1% of a chronic HF population was simultaneously treated with the target doses of GDMT, mostly due to medical reasons [92]. As a result, different algorithms have been proposed which aim at achieving in-hospital implementation of HF treatment as well as rapid titration and escalation of the medical treatment in the outpatient setting [10].

## Conclusion and future considerations

Despite major advances in prevention and medical therapy, HF remains associated with high morbidity and mortality, especially in older and frailer patients. Therefore, a complete, guideline-based treatment is essential, even in HF patients with conditions traditionally associated with a problematic initiation and escalation of the medical HF therapy, such as CKD and arterial hypotension, as the potential adverse effects are overcome by the overall decrease of the absolute risk. Furthermore, since the latest data suggest that the benefit of a combined medical therapy (MRA, ARNI, SGLT2i, beta-blocker) may extend up to a LVEF of 65%, further trials on these subgroups of patients (HFmrEF, HFpEF) are needed to re-evaluate the guideline-directed medical therapy across the HF spectrum. In particular, the use of SGLT2i was recently extended to HFpEF patients, as evidenced by the DELIVER and EMPEROR-preserved trials. Moreover, the indication for other conservative treatments in HF patients, such as the intravenous iron supplementation, has been strengthened in the latest guidelines. Finally, the possible implementation of newer substances in guideline-directed medical practice for HF is anticipated with great expectation.

## Data Availability

Not applicable.
